# Paroxysmal Hæmoglobinuria

**Published:** 1903-10-24

**Authors:** 


### PAROXYSMAL HÆMOGLOBINURIA.

Dr. W. Gilman Thompson [1] reports two cases of this condition which have come under his notice. The first patient was a German, 20 years of age. His father and mother were both markedly neurotic. The first attack of hæmoglobinuria had been noticed when the patient was in his fourth year, and occurred after a sleigh ride. Since that time there had been numerous attacks, mostly in winter-time, and usually brought about by exposure to cold, though they occasionally occurred in warm weather as a result of emotional causes. The paroxysms were quite typical. After a short exposure to moderate cold the hands and feet became stiff, numb, swollen, and cyanosed; there was a general sensation of chilliness, but rarely a definite rigor; there was an intense sensation of weariness, with yawning, severe headache, and abdominal cramps. Itching and burning were felt, and large urticarial wheals appeared upon the face and hands. The temperature rose two or three degrees; there was excessive thirst and pain in the lumbar region. Micturition was frequent, the desire recurring every 10 or 15 minutes, but there was no pain or strangury. In all about a pint of blood-stained urine was voided during the attack, which lasted about one hour, provided that the patient could go at once to bed and get warm. Apart from the paroxysms the patient complained of weakness and inability to apply himself to work, mental or physical. There had never been any jaundice. There was evidence of congenital syphilis, the patient having teeth of the type described by Hutchinson, and having suffered from interstitial keratitis of both eyes. Blood examination failed to show any anæmia. In between the attacks the urine appeared to be normal, except for the presence of phosphates in excess.

The second case was that of an American, 17 years of age. There was a history of insanity on the father's side of the family, and there were some indications that the patient suffered from hereditary syphilis. The first attack of paroxysmal hæmoglobinuria occurred when the patient was three years old. At that time he was observed to have attacks of fever accompanied by jaundice and the passage of dark-coloured urine. It was always noticed by the parents that the attacks were induced by exposure to cold, and that they lasted until the child had become warm again. When the boy was five years old the attacks had become more serious, the urine remaining dark-coloured for a day or two after each paroxysm. It was also noticed that whenever the hands were placed in cold water they became purple, mottled, and numb. At 13 years of age he commenced to have urticarial swelling of the face and hands, epigastric cramps, and bilious vomiting, in addition to the hæmoglobinuria. While under observation an attack was experimentally induced by immersing the patient's hands for two minutes in water at a temperature of 60° F.

In commenting on these two cases Dr. Thompson calls attention to the early age at which the symptoms began, their long duration (fourteen and sixteen years), the prominence of urticaria, œdema and local cyanosis, and the probable existence of hereditary syphilis. He remarks that the only disease with which the condition under discussion is associated with any marked frequency is hereditary syphilis. In approximately one-third of all cases syphilis, either hereditary or acquired, has been noticed, and some cases have been benefited or cured by anti-syphilitic treatment. Removal of the patient to a warm climate during the winter months is usually successful in preventing the recurrence of attacks. If from the circumstances of the patient this change of climate is rendered impossible, antisyphilitic treatment should be instituted and care taken to avoid exposure to cold as far as is possible. Dr. Thompson regards the condition generally as "a profound neurosis, chiefly affecting the vasomotor system, and called into activity by exposure to moderate degrees of cold, by muscular fatigue or mental emotion."

[1] Medical News, Oct. 3.

